# Chronic constant light exposure aggravates high fat diet-induced renal injury in rats

**DOI:** 10.3389/fendo.2022.900392

**Published:** 2022-07-29

**Authors:** Lin Xing, Shanyu Wu, Ying Shi, Fangzhi Yue, Lin Wei, Ryan Russell, Dongmei Zhang

**Affiliations:** ^1^ Department of Endocrinology, Xiangya Hospital, Central South University, Changsha, China; ^2^ Department of Health and Human Performance, College of Health Professions, University of Texas Rio Grande Valley, Brownsville, TX, United States; ^3^ National Clinical Research Center for Geriatric Disorders, Xiangya Hospital, Central South University, Changsha, China

**Keywords:** light pollution, obesity-related kidney disease, circadian disruption, hypoxia-induced factor 1α, prolyl hydroxylase domain

## Abstract

Obesity-related kidney disease is now recognized as a global health issue, with a substantial number of patients developing progressive renal failure and end-stage renal disease. Interestingly, recent studies indicate light pollution is a novel environmental risk factor for chronic kidney disease. However, the impact of light pollution on obesity-related kidney disease remains largely unknown, with its underlying mechanism insufficiently explained. Renal hypoxia induced factor 1α (HIF1α) is critical in the development of glomerulosclerosis and renal fibrosis. The present study explored effects of constant light exposure on high fat diet (HFD) -induced renal injury and its association with HIF1α signal pathway. Thirty-two male Sprague Dawley rats were divided into four groups according to diet (HFD or normal chow diet) and light cycles (light/dark or constant light). After 16 weeks treatment, rats were sacrificed and pathophysiological assessments were performed. In normal chow fed rats, constant light exposure led to glucose abnormalities and dyslipidemia. In HFD fed rats, constant light exposure exacerbated obesity, glucose abnormalities, insulin resistance, dyslipidemia, renal functional decline, proteinuria, glomerulomegaly, renal inflammation and fibrosis. And, constant light exposure caused an increase in HIF1α and a decrease in prolyl hydroxylase domain 1 (PHD1) and PHD2 expression in kidneys of HFD-fed rats. Then, we demonstrated that BMAL1 bound directly to the promoters of PHD1 in mouse podocyte clone 5 cell line (MPC5) by ChIP assays. In conclusion, chronic constant light exposure aggravates HFD-induced renal injuries in rats, and it is associated with activation of HIF1α signal pathway.

## Introduction

Obesity has long been associated with chronic kidney disease (CKD) (1). The characteristic features of obesity-related kidney disease include glomerular hypertrophy, thickening of the glomerular basement membrane, mesangial matrix expansion, and increased renal inflammation (2). Although most patients with obesity-related kidney disease have stable or slowly progressive proteinuria, up to one-third develop progressive renal failure and end-stage renal disease (ESRD) (3). With the rapid increase in obesity prevalence worldwide, obesity-related kidney disease is becoming a prominent cause of ESRD (4). Nonetheless, the mechanisms responsible for progression of the disease and its underlying pathogenesis are not yet well understood.

Roles of environmental risk factors, such as light pollution, in CKD have gained increasing interest in recent years (5). Light pollution is caused by excessive and inappropriate introduction of artificial light by humans into indoor and outdoor environments (6). With rapid urbanization and economic development, light pollution has inevitably become globalized (7). Light pollution contributes to several human pathophysiologies, though has not been examined as a risk factor for kidney diseases. However, a recent epidemiological study demonstrated that shift-workers (in a sense, they expose to high levels of artificial light at night in the workplace) had increased CKD risk (8), thus raising the possibility that prolonged exposure to light (light pollution) might play a role in kidney diseases.

As the most prominent chronobiology disruptor, light pollution is considered detrimental to human health because of its disruptive effect on circadian rhythms (9). The circadian clock is an endogenous timing-system that enable living organisms to coordinate their behavior and physiology with daily environmental changes (10). It is generated by a series of physiological clocks, the master clock located in the suprachiasmatic nucleus, and other physiological clocks located in peripheral tissues, including kidney (11). The kidney circadian clock contributes to the regulation of renal functions such as renal plasma flow, glomerular filtration rate and tubular transport activities (12). There appears to be a connection between circadian disruption and CKD. For example, in patients with mild to moderate CKD, lower eGFR was associated with shorter sleep duration, greater sleep fragmentation and later timing of sleep (13). As noted in animal models, *Clock* KO mice developed more severe kidney fibrosis upon ureteral obstruction (14).

These observations indicate that circadian disruption affects the development of CKD and light pollution may theoretically play a role in the progression of obesity related kidney disease. Moreover, links between obesity and light pollution have been well reported. Epidemiological evidence suggests shift workers are more prone to weight gain and are susceptible to obesity-linked pathophysiologies (15). Further, mice exposed to nighttime light are prone to obesity (16). We previously found that constant light exposure aggravated obesity and visceral adiposity in rats fed with high fat diet (HFD) (17). However, few studies have observed the effects of constant light exposure (i.e. light pollution) on obesity related kidney disease.

Hypoxia-inducible factor 1α (HIF1α) is a master signal mediator of hypoxic responses. Under normoxic conditions, HIF1α protein is rapidly hydroxylated by a group of prolyl hydroxylase domain (PHD) enzymes – PHD1, PHD2 and PHD3 – and are degraded by proteasomes (18). Conversely, during hypoxia HIF1α is not hydroxylated, which results in increased net levels of HIF1α and subsequent activation of HIF1α target gene expression, such as transforming growth factor-β (TGF-β), connective tissue growth factor (CTGF) and vascular endothelial growth factor (VEGF), and plays a critical role in glomerulosclerosis and renal fibrosis in CKD (19). Genetic ablation of proximal tubule epithelial HIF1α appears to impede the development of kidney fibrosis in unilateral ureteral obstruction in mice (20). In mouse models of diabetic nephropathy, downregulation of HIF1α is associated with improved renal function, reduced proteinuria, and glomerulosclerosis (21). Glomerulosclerosis is one of several pathological characters of obesity-related kidney disease, with renal fibrosis being the final common pathway between kidney diseases and ESRD. However, the role of HIF1α in obesity-related kidney disease remains largely unexplored.

To investigate the impact of chronic constant light exposure on the development of obesity-related kidney disease, we utilized an HFD-induced obesity model, and explored effects of constant light exposure on HFD-induced renal injury and its association with HIF1α signal pathway.

## Materials and methods

### Animals and experimental design

All animal experimental procedures were approved by the Medical Ethics Committee of Central South University and performed in accordance with the guidelines established by the committee (No.2018sydw184). Thirty-two male SD rats (six-week-old, weighing 250–270g) obtained from the Hunan Slac Jinda Laboratory Animal Company (Changsha, China), were housed under controlled conditions (22 ± 2°C, 40 ~ 50% humidity) with free access to water and food. After acclimating for one week, rats were randomly divided into four groups (n=8 per group) and housed in two separate rooms (1): ND-LD group, rats were fed on a normal chow diet (ND, fat 12%, protein 22%, carbohydrate 66%, 3.48 kcal/g) and exposed to a standard 12:12 hour light/dark (LD) cycle (light:7am-7pm, 200lux; dark: 7pm-7am) (2), ND-LL group, rats were fed on a normal chow diet and exposed to constant light (LL) (200 lux) (3), HFD-LD group, rats were fed on a high-fat diet (HFD, fat 37%, protein 17%, carbohydrate 46%, 4.40 kcal/g) and exposed to standard 12:12 hour LD cycle, and (4) HFD-LL group, rats were fed on an HFD and exposed to constant light (LL) (200 lux). The light source was regular natural white fluorescent light tubes with a wavelength range of 400~560 nm.

Body weight was recorded weekly for all animals throughout the experiment. Glucose and insulin tolerance tests were performed at the end of the 14^th^ week and the beginning of the 15^th^ week of intervention. Twenty four-hour urine collection using a metabolic cage was performed at the 8^th^ and 15^th^ week of intervention.

At the end of the 16^th^ week of intervention, the rats were sacrificed between 8:00 am and 11:00 am in two consecutive days by intraperitoneal injection (a single dose) of pentobarbital sodium (100 mg/kg) accompanied by isoflurane inhalation to maintain anesthesia of the animal throughout the surgical protocols. Blood samples were collected from the inferior vena cava, and the kidneys were harvested immediately for histological study and biochemical analysis. Perirenal and epididymal adipose tissue weights were measured to assess visceral fat mass. Kidney tissues were immediately frozen in liquid nitrogen and stored at -80°C for RNA extraction and western blot, or fixed in 10% neutral formalin and 2.5% glutaraldehyde respectively for paraffin sections and ultrastructural examination by transmission electron microscope.

### Glucose and insulin tolerance tests

Briefly, the rats were fasted overnight and injected intraperitoneally with 50% D-glucose (2.0 g/kg, kelun, Hunan, China) or insulin (0.75 IU/kg, Novolin R, Novo Nordisk, Denmark). Blood glucose levels were measured at 0, 15, 30, 60 and 120 minutes *via* tail bleed with the Accu Check Advantage system (Roche Diagnostics, Mannheim, Germany). Plasma glucose concentrations following the glucose or insulin loading was expressed as total area under the curve for glucose (AUC) using the trapezoidal rule.

### Serum analysis

Serum triglycerides (TG), total cholesterol (TC), low-density lipoprotein cholesterol (LDL-C), high-density lipoprotein cholesterol (HDL-C), free fatty acid (FFA), blood urea nitrogen (BUN), and creatinine levels were measured using commercially available reagents (Serotec Co., Sapporo, Japan).

Serum tumor necrosis factor-α (TNF-α) and interleukin 6 (IL-6) were measured using respective commercial rat-specific enzyme-linked immunosorbent assay (ELISA) kits (Cusabio, Wuhan, China).

### Urinary albumin concentration

Urinary albumin excretion was determined using a commercially available ELISA kit (Cusabio, Wuhan, China) according to the manufacturer’s instructions and was expressed as total albumin excretion in 24 h.

### Renal histopathology

Renal tissues were fixed in 10% neutral formalin, embedded in paraffin, serially sectioned (3 µm), and stained with hematoxylin-eosin (HE), periodic acid–Schiff (PAS), and Masson’s trichrome solution as previously described (22). HE staining was used to assess the level of renal injury by calculating glomerular injury scores on a blinded basis (23). More than 10 consecutive fields were examined at 400× magnification. The score index in each rat was expressed as a mean value of all scores obtained. Mesangial matrix expansion was defined by PAS-positive area in the mesangial region. Masson’s trichrome staining was used to evaluate extent of interstitial fibrosis and glomerulosclerosis, which was quantified by Image J (National Institutes of Health, Bethesda, MD).

### Transmission electron microscopy

Renal tissues were promptly cut into 1 mm^3^ pieces and fixed in 2.5% glutaraldehyde, post-fixed in 1% osmium tetroxide, dehydrated in graded alcohols, and embedded in Epon. Ultrathin sections (200–400 Å) were cut on nickel grids, stained with uranyl acetate and lead citrate, and examined using a transmission electron microscope (Hitachi H-7500, Chiyoda-ku, Tokyo, Japan) (24).

### Quantitative real-time polymerase chain reaction

Total kidney RNA was extracted using TRIzol reagent (Life Technologies Corporation, Woburn, MA, USA) and reverse transcribed using HiFiScript cDNA synthesis kit (Cowin Bioscience, Jiangsu, China). The PCR primers (sequences were listed in **Supplementary Table 1**) were obtained from Shanghai Bioshang biotechnology company, and qRT-PCR was performed on a Rotor-Gene 6000 instrument (Corbett Life Science, Mortlake, NSW, Australia). The cycling program was 95°C for 10 minutes followed by 40 cycles of 95°C for 15 seconds, and 60°C for 1 minute. The relative abundance of the target genes was normalized to β-actin as an internal control.

### Western blot analysis

Renal tissues were homogenized in lysis buffer (Cowin Bioscience, China), and the protein concentrations were measured using a BCA Protein Assay Kit (Biosharp life science, China). Protein samples (20 μg/lane) were then subjected to SDS-PAGE electrophoresis, transferred to polyvinylidene fluoride (PVDF) membranes (Millipore, Billerica, MA, USA), and blocked in 5% non-fat dry milk at room temperature for 90 min. The membranes were incubated with anti-TGF-β (1:1000 dilution, Abcam, Cambridge, UK), anti-HIF1α (1 μg/mL, R&D systems, Minneapolis, MN, USA), anti-PHD1 (1:1000 dilution, Proteintech, Wuhan, China), anti-PHD2 (1:500 dilution, Proteintech, Wuhan, China), and anti-β-actin (1:5000 dilution, Proteintech, Wuhan, China) at 4°C overnight and then incubated with horseradish peroxidase (HRP)-conjugated secondary antibodies (Proteintech, Wuhan, China) at room temperature for 90 min. The immune reactivity was detected by an enhanced chemiluminescence reagent (Biosharp life science, Hefei, China).

### Immunohistochemistry staining

For IHC, paraffin-embedded kidney sections (3 µm) were deparaffinized, rehydrated, blocked, and incubated with various primary antibodies, including anti-CD68 (1:500, Boster Biological Technology, Wuhan, China), anti-Nephrin (1:100, Affinity Biosciences, Cincinnati, OH, USA), anti-TGF-β (1:500, Abcam, Cambridge, UK), and anti-HIF1α (5 μg/mL, R&D systems, Minneapolis, MN, USA) overnight at 4°C. Sections were then washed with PBS containing 0.1% Triton and incubated with HRP-conjugated secondary antibody (ZSGB Biotechnology, Beijing, China) for 1 h at room temperature. After the final wash with PBS/triton, sections were stained with DAB (ZSGB Biotechnology, Beijing, China) substrate and hematoxylin. The images of stained sections were acquired by bright field microscope, and quantitative analysis of positive staining areas (%) in images was done using ImageJ (National Institutes of Health, Bethesda, MD). Identical staining without the primary antibody was used as a negative control.

### Cell culture

MPC5, a conditionally immortalized mouse podocyte clone 5 cell line was purchased from Cell Bank of the Chinese Academic of Sciences (Shanghai, China) and cultured as previously described (25). Briefly, cells were cultured in Dulbecco’s Modified Eagle Medium (DMEM, Gibco, USA) supplemented with 10% fetal bovine serum (FBS; Gibco, USA) in a humidified atmosphere at 37°C with 5% CO2. Cells were passaged at 33°C and treated with 10U/mL mouse recombinant interferon gamma (IFN-γ). To induce podocyte differentiation, the temperature was increased to 37°C and cells were cultured in medium without IFN-γ for 14 days, after which subsequent experiments were performed.

### Chromatin immunoprecipitation assays

The ChIP assay was performed using a commercial kit (ab500, Abcam, Cambridge, UK) according to the manual instructions. MPC5 cells were fixed with 1% formaldehyde for 10 min at room temperature (25°C) for protein-DNA crosslinking. Subsequently, cells were quenched by incubation with 125 mM glycine for 5 min. After washing with cold PBS, the cells were pelleted at 500 × g for 5 min at 4°C. The pellets were resuspended and lysed by adding 1 mL of cold lysis buffer containing protease inhibitor cocktail. Cell lysates were then sonicated with Misonix S3000 Sonicator (Farmingdale, USA). After centrifugation at 14,000 × g for 5 min at 4°C, chromatin supernatants were diluted with cold ChIP dilution buffer. The antibody against BMAL1 (1µg, 14268-1-AP, Proteintech, Wuhan, China), or normal IgG (1µg, ab171810, Abcam, Cambridge, UK) was added and incubated at 4°C overnight. The precipitates were washed. The chromatin complexes were eluted. The DNA was purified and used as a template for qPCR. The sequences of primers used for ChIP assays are listed in **Supplementary Table 1**.

### Statistical analysis

Statistical analysis was carried out using GraphPad Software (San Diego, CA). All values are presented as mean ± SEM. The significance of differences was determined by the use of a one-way or two-way ANOVA followed by a Bonferroni *post-hoc* analysis where appropriate. Differences were considered significant when *p<*0.05.

## Results

### Effects of constant light exposure on body weight and metabolic parameters

**Figure 1:**
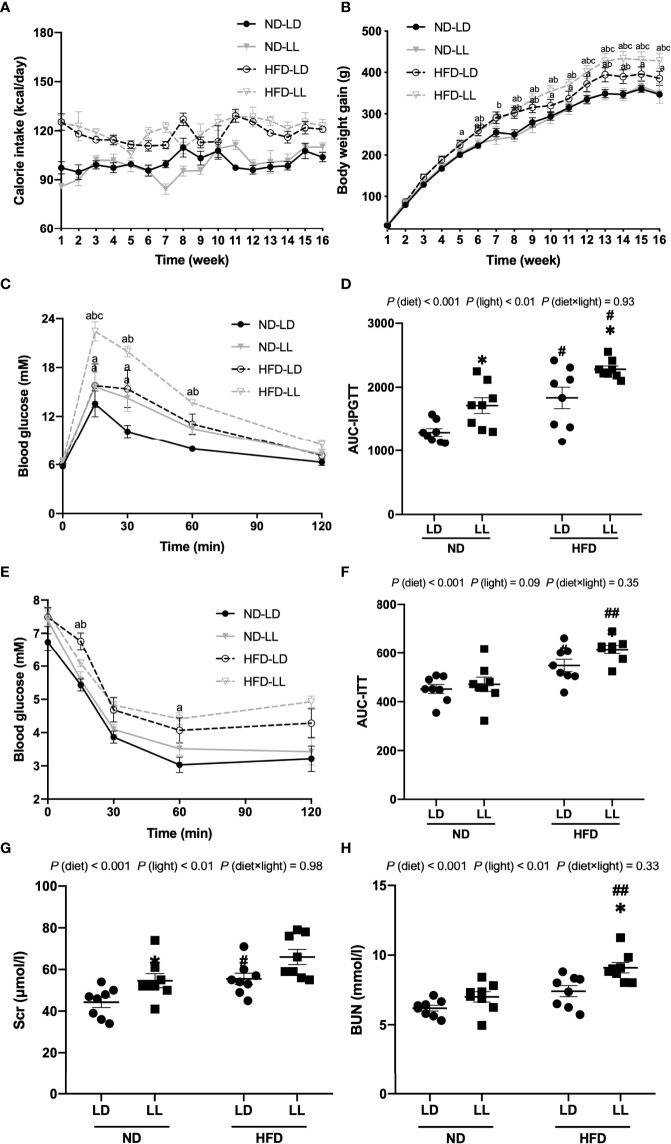
Effects of constant light on calorie intake, body weight gains, intraperitoneal glucose tolerance test (IPGTT), insulin tolerance test (ITT) and renal function in rats. **(A)** Changes of calorie intake. **(B)** Changes of body weight gain. **(C)** IPGTT. **(D)** Area under the curve (AUC) of IPGTT. **(E)** ITT. **(F)** AUC of ITT. **(G, H)** Serum creatinine (Scr) and blood urea nitrogen (BUN) at the end of the experiment. Values represent mean ± SEM (n=8). Differences were determined using either a one-way ANOVA **(A–C, E)** or a two-way ANOVA followed by a Bonferroni post hoc analysis **(D, F–H)**. ^a^ *p*<0.05 *vs.* ND-LD group, ^b^ *p*p<0.05, *vs.* ND-LL group, ^c^ *p*p<0.05, *vs.* HFD-LD group. ^#^ *p*<0.05, ^##^ *p*<0.01, *vs.* ND counterpart. *p<0.05, *vs.* LD counterpart. *p* (diet), main effect of diet; *p* (light), main effect of light; *p* (light × diet), interaction effect of light and diet.

There were no significant differences in caloric intake or weight gains during 16 weeks of feeding between the ND-LD and ND-LL groups (**Figures 1A, B**). HFD-LL group had increased body weights than the HFD-LD group from the 12^th^ to the 16^th^ week of HFD feeding (**Figures 1A, B**). At the end of experiment, kidney weight/body weight was lower in HFD-fed rats vs. ND-fed rats [*p* (diet) <0.001] (**Table 1**). Body weight, lee’s index, and visceral fat mass were significantly higher in the HFD-LL group vs. the HFD-LD group, with the significant main effects of both diet (*p*<0.001) and interactive effects between diet and light (*p*<0.05) (**Table 1**). In ND-fed rats, constant light exposure caused an increase in serum TC levels. In HFD-fed rats, constant light exposure led to a comparable increase in serum TC and LDL-C levels. (**Table 1**).

**Table 1:** Characteristics of rats in each group at the end of experiment.

Parameters	Group				2-Way ANOVA Statistics
	ND-LD	ND-LL	HFD-LD	HFD-LL	
n	8	8	8	8	
Body weight (g)	600.50 ± 6.85	579.90 ± 16.90	641.96 ± 17.52^a,b^	695.64 ± 16.02^a,b,c^	*p* (diet) <0.001 *p* (diet × light) < 0.05
Lee’s index	295.68 ± 2.59	290.8 ± 2.58	309.48 ± 3.18^a,b^	319.67 ± 1.90^a,b,c^	*p* (diet) <0.001 *p* (diet × light) < 0.05
Visceral fat mass (g)	31.57 ± 4.79	28.46 ± 3.40	50.74 ± 3.25^a,b^	66.16 ± 2.65^a,b,c^	*p* (diet) <0.001 *p* (diet × light) < 0.05
Visceral fat mass/body weight ratio (×10^-2^)	5.59 ± 0.48	4.99 ± 0.61	7.73 ± 0.76^a,b^	9.27 ± 1.07^a,b,c^	*p* (diet) <0.001
Kidney weight (g)	3.57 ± 0.10	3.45 ± 0.13	3.64 ± 0.10	3.74 ± 0.14	ns
Kidney weight/body weight ratio (×10^-3^)	6.05 ± 0.13	6.13 ± 0.24	5.70 ± 0.08^a^	5.55 ± 0.15^a,b^	*p* (diet) <0.001
TG (mmol/L)	0.77 ± 0.12	0.60 ± 0.14	0.92 ± 0.09	0.84 ± 0.18	ns
TC (mmol/L)	1.42 ± 0.09	1.59 ± 0.11^a^	1.85 ± 0.19^a,b^	2.16 ± 0.19^a,b,c^	*p* (diet) < 0.001 *p* (light) <0.05
HDL-C (mmol/L)	0.85 ± 0.06	0.90 ± 0.06	0.63 ± 0.04^a,b^	0.66 ± 0.05^a,b^	*p* (diet) < 0.001
LDL-C (mmol/L)	0.49 ± 0.05	0.56 ± 0.04	0.84 ± 0.09^a,b^	1.05 ± 0.07^a,b,c^	*p* (diet) <0.001 *p* (light) <0.05
FFA (mmol/L)	0.46 ± 0.06	0.43 ± 0.08	0.45 ± 0.05	0.48 ± 0.03	ns
TNF-α (pg/mL)	9.40 ± 0.11	9.62 ± 0.07	13.40 ± 0.30^a,b^	16.67 ± 0.33^a,b,c^	*p* (diet) <0.001 *p* (light) <0.001 *p* (diet × light) <0.001
IL-6 (pg/mL)	26.82 ± 1.65	40.26 ± 2.75^a^	46.26 ± 2.21^a^	70.01 ± 2.10^a,b,c^	*p* (diet) <0.001 *p* (light) <0.001 *p* (diet × light) < 0.05

Values are mean ± standard error of the mean (SEM). Differences were determined using a two-way ANOVA followed by a Bonferroni post hoc analysis. ^a^p<0.05, vs. ND-LD group, ^b^p<0.05, vs. ND-LL group, ^c^p<0.05 vs. HFD-LD group. p (diet), main effect of diet; p (light), main effect of light; p (light × diet), interaction effect of light and diet. Lee’s index, evaluation for the obese degree of rats; TG, triglyceride; TC, total cholesterol; HDL-C, high-density lipoprotein cholesterol; LDL-C, low-density lipoprotein cholesterol; FFA, free fatty acid; TNF-α, tumor necrosis factor-alpha; IL-6, interleukin-6.

### Effects of constant light exposure on glucose homeostasis

In ND-fed rats, ND-LL animals had significantly higher blood glucose levels at 15min and 30 min than the ND-LD group during the IPGTT. AUC for IPGTT was higher in ND-LL group than that of ND-LD group [*p* (diet) <0.001; *p* (light) <0.01] (**Figures 1C, D**). As for ITT, there were no statistical differences in blood glucose levels or AUC-ITT between ND-LD and ND-LL groups (**Figures 1E, F**).

IPGTT and ITT revealed impaired glucose tolerance and greater insulin resistance in HFD-fed rats. Compared with the HFD-LD group, HFD-LL rats had elevated blood glucose levels at 15 min during IPGTT and higher AUC-IPGTT (**Figures 1C, D**). ITT showed greater insulin resistance in HFD-LL vs. HFD-LD group [*p* (diet) <0.001] (**Figures 1E, F**).

### Effects of constant light exposure on proteinuria, renal function and glomerulopathy

**Figure 2:**
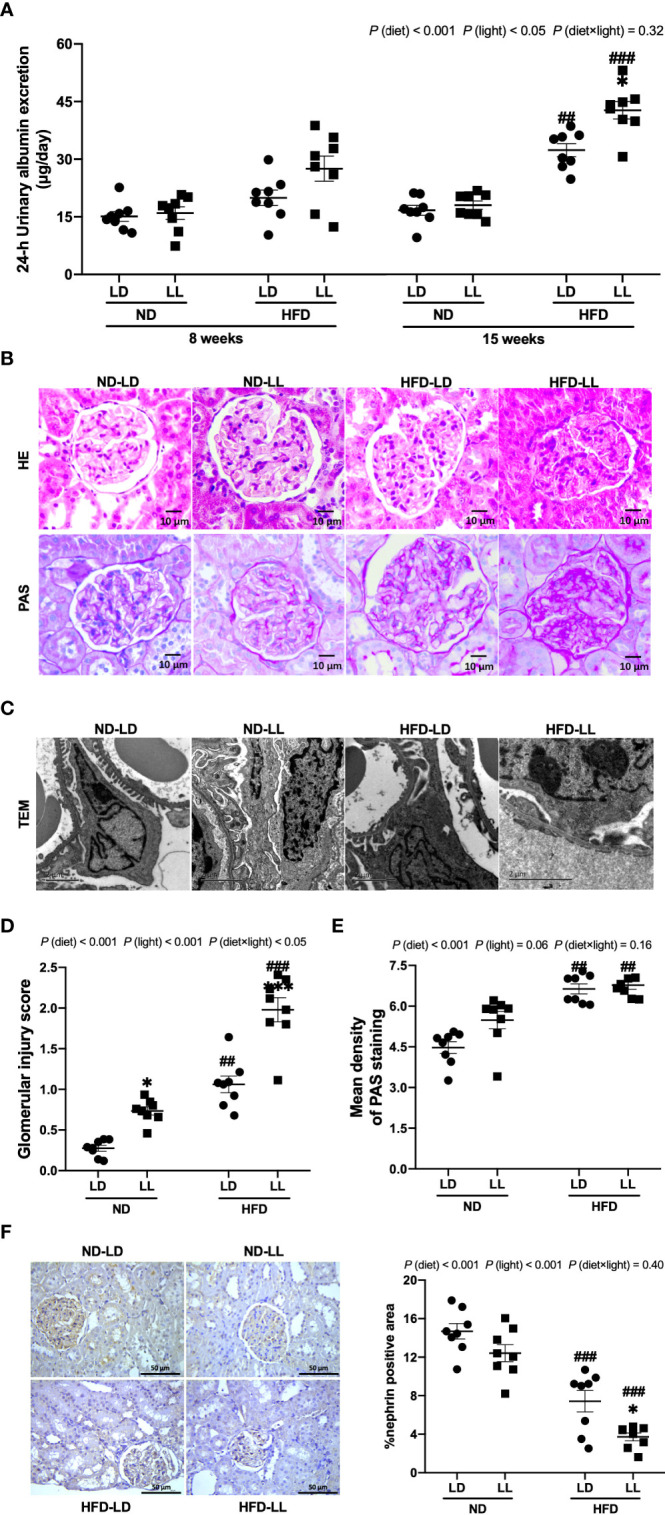
Effects of constant light exposure on urinary albumin exertion and glomerulopathy in rats. **(A)** 24-h urinary albumin excretion at 8^th^ and 15^th^ week of experiment in rats. **(B)** Representative images of HE staining and PAS staining of glomeruli (magnification, 40×) as indicated. Scale bar=10μm. **(C)** Representative transmission electron microscopic (TEM) images of glomeruli as indicated. Scale bar = 2μm. **(D)** Glomerular injury scores. **(E)** Densitometric analyses of PAS staining. **(F)** Renal expression of nephrin by IHC and semi-quantification analysis. Values represent mean ± SEM (n=8 for **A, D–F**). Differences were determined using a two-way ANOVA followed by a Bonferroni post hoc analysis. ^##^ *p*<0.01, ^###^ *p*<0.001, *vs.* ND counterpart. *p<0.05, **p<0.01, *vs.* LD counterpart. *p* (diet), main effect of diet; *p* (light), main effect of light; *p* (light × diet), interaction effect of light and diet.

At the 8^th^ week of experiment, there was no significant difference in urinary albumin excretion among groups. At the 15^th^ week, HFD-fed groups had significantly greater excretion of proteinuria than ND-fed rats. Urinary albumin excretion was significantly higher in the HFD-LL group vs. the HFD-LD group [*p* (diet) <0.001; *p* (light) <0.05] (**Figure 2A**). At the end of experiment, ND-LL, HFD-LD and HFD-LL groups had higher concentration of serum creatinine than the ND-LD group. In HFD-fed rats, HFD-LL group displayed higher serum BUN than HFD-LD group [*p* (diet) <0.001; *p* (light) <0.01] (**Figures 1G, H**).

Pathologically, ND-LL ras manifested glomerular hypertrophy (**Figure 2B**), and had slightly higher glomerular injury scores than ND-LD rats (**Figure 2D**). In HFD-fed rats, glomerular mesangial expansion and glomerular basement membrane thickening were observed. And these changes were more severe in HFD-LL rats vs. HFD-LD rats (**Figure 2B**). In HFD-fed rats, transmission electron microscopy revealed irregular shapes with flattened foot processes and some areas of effacement in podocytes, and these changes were aggravated in HFD-LL rats (**Figure 2C**). In line, HFD resulted in higher glomerular injury scores, with even higher glomerular injury scores in HFD-LL *vs.* HFD-LD rats (**Figure 2D**). Main effects of diet (*p*<0.001) and light (*p*<0.01), as well as diet × light interactive effect (*p*<0.05) were observed for glomerular injury scores. In addition, a significant elevation of PAS-positive matrix was also noted in HFD-fed groups *vs.* ND-fed groups [*p* (diet) <0.001] (**Figure 2E**). Expression of nephrin, the key structural molecule of the glomerular filtration barrier (26), was decreased in HFD-fed rats, with HFD-LL rats displayed even lower expression of nephrin than HFD-LD rats [*p* (diet)<0.001; *p* (light) <0.01] (**Figure 2F**).

Visceral fat was positively correlated with urinary albumin concentration (*r* = 0.812, *p*<0.001) and glomerular injury score in HFD-fed rats (*r* = 0.806, *p*<0.001). However, there were no relationships noted between visceral fat mass and urinary albumin concentration, or glomerular injury scores in ND-fed rats (*p*>0.05).

### Effects of constant light exposure on renal inflammation in rats

**Figure 3:**
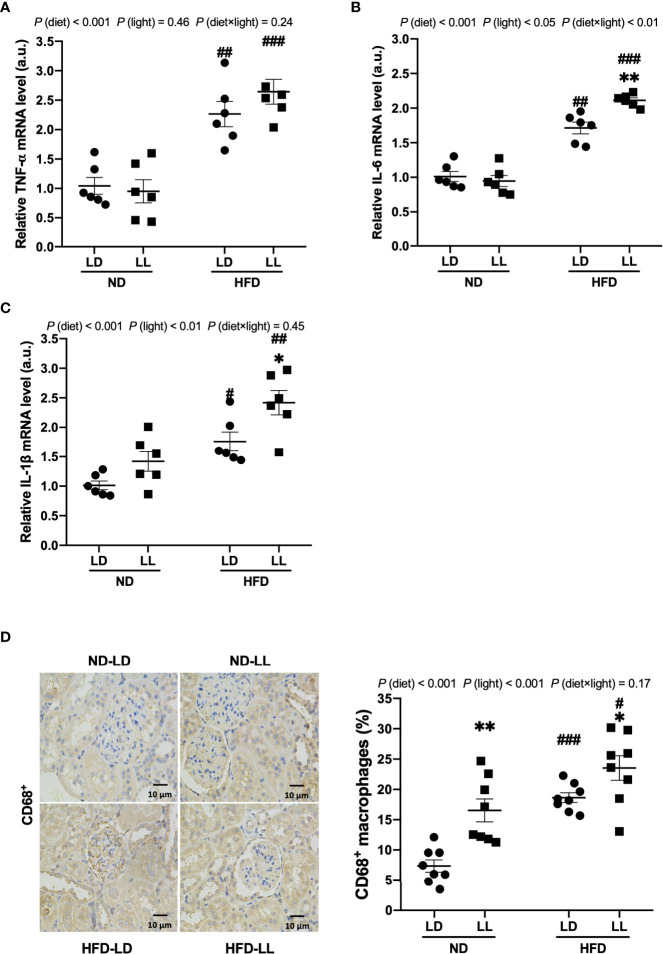
Constant light exposure promotes renal inflammation in HFD-fed rats. **(A–C)** Renal mRNA expression of *TNF-α*, *IL-6* and *IL-1β* in rats. **(D)** IHC staining of CD68 and quantification of CD68^+^ macrophages. Values represent mean ± SEM (n=6 for **A–C**, n=8 for **D**). Differences were determined using a two-way ANOVA followed by a Bonferroni post hoc analysis. ^#^ *p*<0.05, ^##^ *p*<0.01, ^###^ *p*<0.001, vs. ND counterpart. *p<0.05, **p<0.01, vs. LD counterpart. *p*(diet), main effect of diet; *p* (light), main effect of light; *p* (light × diet), interaction effect of light and diet.

HFD-fed rats had significantly higher serum TNF-α and IL-6 levels than ND-fed rats. Constant light exposure further increased serum TNF-α and IL-6 levels in HFD-fed rats, with significant main effects of diet (*p*<0.001) and light (*p*<0.001) noted, as well as an interactive effect between diet and light (*p*<0.05) (**Table 1**). Renal mRNA expressions of *IL-6* and *IL-1β* were significantly higher in the HFD-LL vs. HFD-LD group (**Figures 3A**–**C**). Main effects of diet (*p*<0.001) and light (*p*<0.05), as well as interactive effect between diet and light (*p*<0.01) were observed for mRNA levels of *IL-6* gene (**Figure 3B**). CD68 is regarded as a marker of macrophage infiltration (27). A significantly higher number of CD68-positive macrophages was observed in HFD-fed rats *vs.* ND-fed rats. In ND-fed rats, ND-LL group had increased infiltration of CD68-positive cells than ND-LD group. Similarly, in comparison to the HFD-LD group, renal CD68-positive macrophages in the HFD-LL group was significantly higher [*p* (diet) <0.001; *p* (light) <0.001] (**Figure 3D**).

### Effects of constant light exposure on renal fibrosis in rats

**Figure 4:**
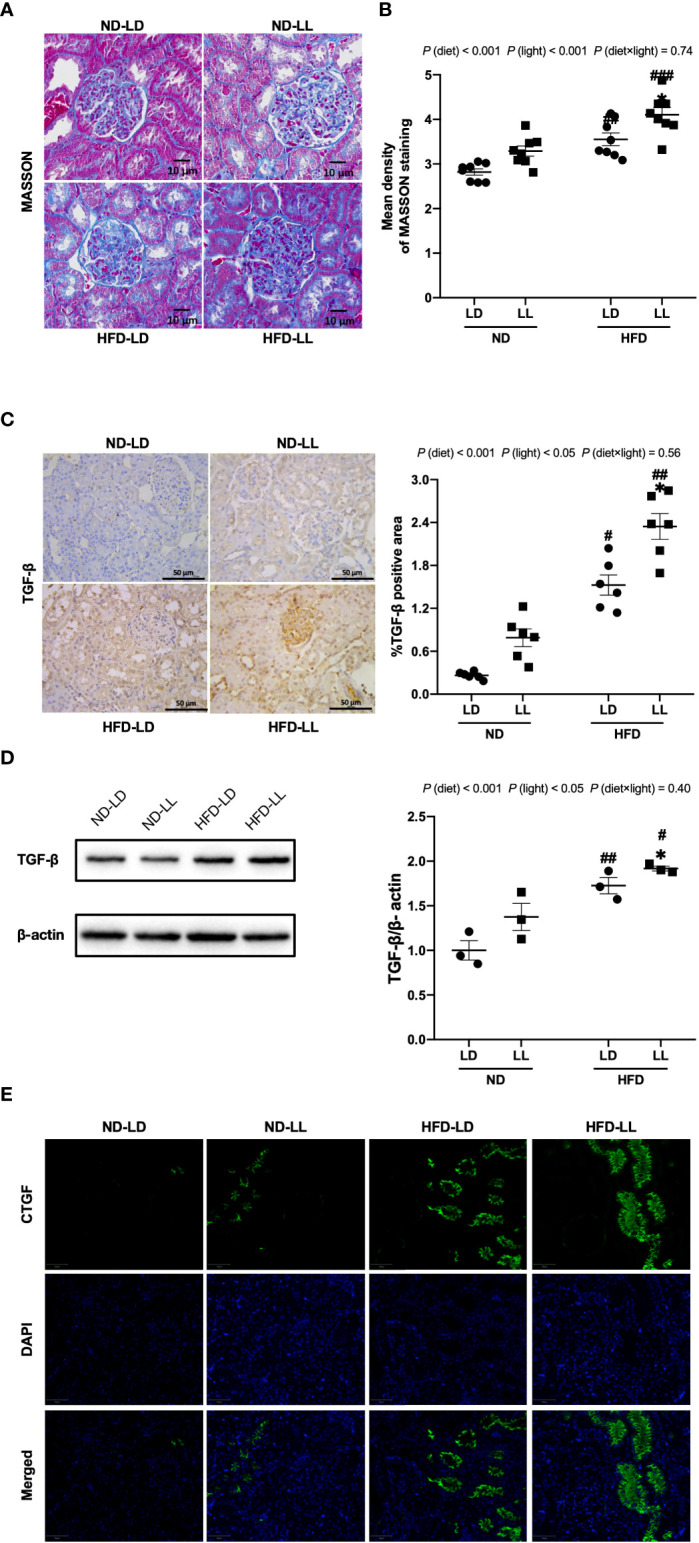
Effects of constant light on renal fibrosis in rats. **(A)** Renal masson’s trichrome staining (40×). Scale bar=10μm. **(B)** Densitometric analyses of Masson’s trichrome staining. **(C)** IHC staining of renal TGF-β and semi-quantification analysis. **(D)** Western blot assays of TGF-β expression in kidney tissues. **(E)** Immunofluorescence staining of CTGF in kidney tissues. Values represent mean ± SEM (n=8 for **B**, n=6 for **C**, n=3 for **D**). Differences were determined using a two-way ANOVA followed by a Bonferroni post hoc analysis. ^#^ *p*<0.05, ^##^ *p*<0.01, ^###^ *p*<0.001, *vs.* ND counterpart. *p<0.05, *vs.* LD counterpart. *p* (diet), main effect of diet; *p* (light), main effect of light; *p* (light × diet), interaction effect of light and diet.

Masson’s trichrome staining illustrated marked fibrotic lesions in the kidneys of HFD-fed rats. These lesions were more severe in HFD-LL rats *vs.* HFD-LD rats (**Figure 4A**). Consistent with this, densitometric analyses showed that in the HFD-LL group, the positive area of Masson’s trichrome staining was significantly more concentrated than the HFD-LD group [*p* (diet) <0.001; *p* (light) <0.001] (**Figure 4B**). TGF-β, with its target gene CTGF plays a key role in the development of renal fibrosis (28). We found significantly greater expression of TGF-β in renal tissue of HFD-fed rats, while the HFD-LL group had higher expression of TGF-β than the HFD-LD group [*p* (diet) <0.001; *p* (light) <0.05] (**Figure 4C**). These findings were further verified by western blots [*p* (diet) <0.001; *p* (light) <0.05] (**Figure 4D**). In addition, HFD feeding induced a marked increase in CTGF expression, with even higher expresions in the HFD-LL *vs.* HFD-LD groups (**Figure 4E**).

Collectively, these data demonstrated that constant light exposure exacerbates renal dysfunction, proteinuria, glomerulopathy, renal inflammation, and fibrosis in HFD-fed rats.

### Effects of constant light exposure on renal expression of HIF1α and PHD proteins in rats

**Figure 5:**
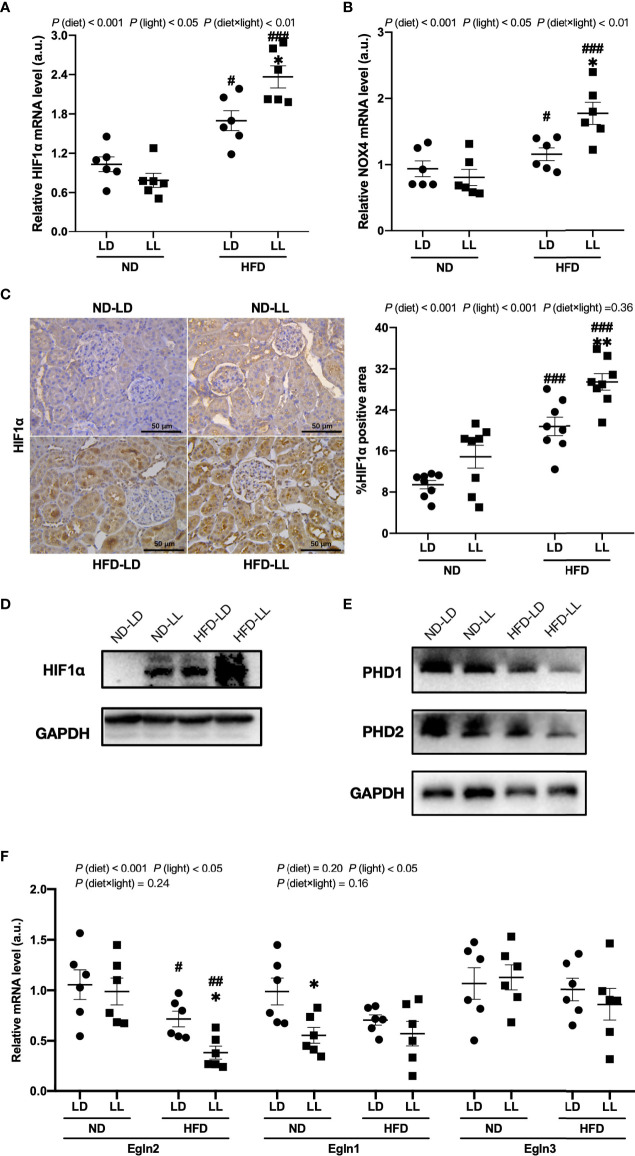
Effects of constant light exposure on renal expression of HIF1α, NOX4 and PHD. **(A, B)** qPCR analysis of mRNA levels of *HIF1α* and *NOX4* in renal tissues of rats. **(C)** IHC staining of renal HIF1α and semi-quantification analysis. **(D)** Western blot assays of HIF1α expression in renal tissues. **(E)** Western blot assays of PHD1 and PHD2 expression in the renal tissues. **(F)** qPCR analysis of mRNA levels of *PHD1 (Egln2)*, *PHD2 (Egln1)*, and *PHD3 (Egln3)* in the kidney. Values represent mean ± SEM (n=6 for **A, B** and **F**, n=8 for **C**). Differences were determined using a two-way ANOVA followed by a Bonferroni post hoc analysis. ^#^ *p*<0.05, ^##^ *p*<0.01, ^###^ *p*<0.001, *vs.* ND counterpart. *p<0.05, **p<0.01, *vs.* LD counterpart. *p* (diet), main effect of diet; *p* (light), main effect of light; *p* (light × diet), interaction effect of light and diet.

The HIF1α pathway is a key regulator of renal fibrosis (29). NADPH oxidase 4 (NOX4) is the major NADPH isoform in kidney (30). Evidence shows that Nox4 interplays with HIF1α and plays a critical role in various renal diseases (31–33). In ND-fed rats, *HIF1α* and *NOX4* mRNA expression showed no significant difference between ND-LL and ND-LD group (**Figures 5A, B**). ND-LL group had increased HIF1α than ND-LD group at protein level (**Figures 5C, D**). Among HIF1α hydroxylation enzymes, *PHD2 (Egln1)* mRNA expression were significantly lower in ND-LL group *vs.* ND-LD group.

In comparison to the ND-LD group, we found a significant increase of *HIF1α* and *NOX4* mRNA expression in HFD-fed groups. Further, these elevated expressions were exacerbated by constant light exposure in HFD-fed rats (**Figures 5A, B**). There were significant main effects of diet (*p <*0.001) and light (*p*<0.05), as well as diet × light interactive effect (*p*<0.01) for both *HIF1α* and *NOX4* mRNA expression (**Figures 5A, B**). The upregulation of HIF1α protein expression was verified by IHC and WB assays (**Figures 5C, D**). Renal mRNA expressions of *PHD1 (Egln2)* and *PHD2 (Egln1)* were significantly decreased in HFD-fed rats, which was also verified by WB assays. Further, there was a significant decrease in renal mRNA expression of PHD1 (*Egln2)* in HFD-LL group vs. HFD-LD group [*p* (diet) <0.001; *p* (light) <0.05] (**Figures 5E, F**).

### Changes of renal circadian clock genes

**Figure 6:**
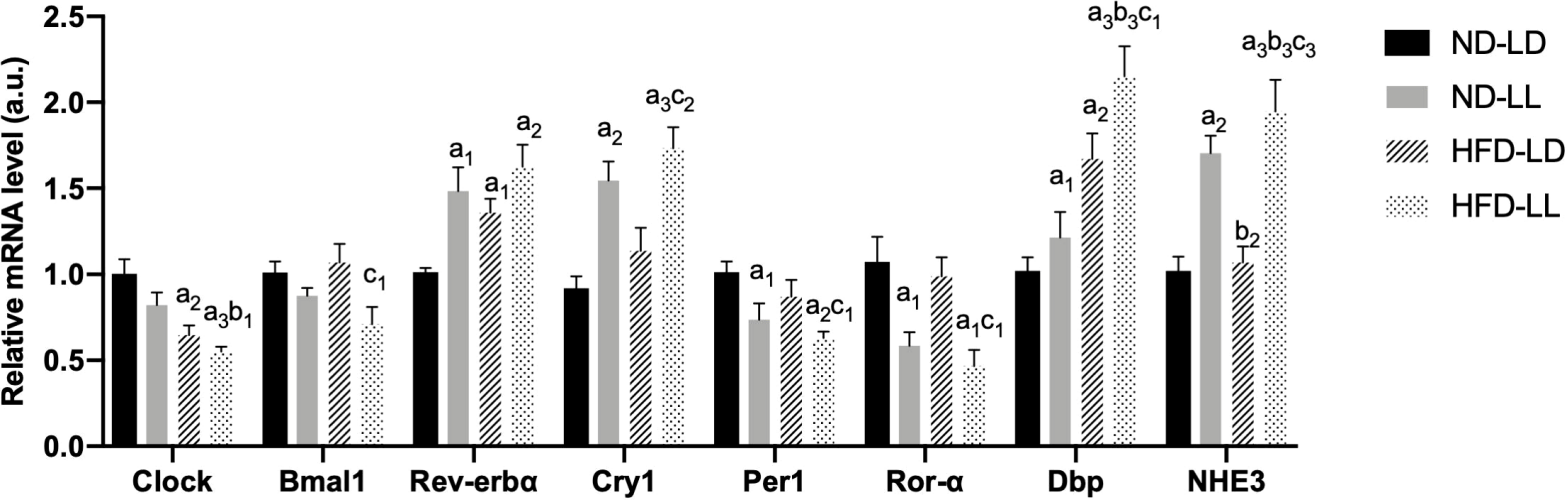
Changes in expression of clock genes in kidneys of rats. Quantitative PCR analysis of mRNA levels of renal circadian clock genes respectively. Values represent mean ± SEM (n=6-8). Differences were determined using a one-way ANOVA followed by a Bonferroni post hoc analysis. ^a1^ *p*<0.05, ^a2^ *p*<0.01, ^a3^ *p*<0.001 vs. ND-LD group; ^b1^ *p*<0.05, ^b2^ *p*<0.01, ^b3^ *p*<0.001 *vs.* ND-LL group; ^c1^ *p*<0.05, ^c2^ *p*<0.01, ^c3^ *p*<0.001 *vs.* HFD-LL group. Bmal1, brain and muscle ARNT-like protein 1; Per1, period circadian regulator 1; Cry1, cryptochrome circadian regulator 1; Ror-α, RAR-related orphan receptor-α; DBP, D site albumin promoter binding protein; NHE3, Na^+^/H^+^ exchanger 3.

Renal mRNA expression of clock genes at the time of sacrifice was assessed by real-time PCR. Increased *Rev-erb*, *Cry1*, *Dbp*, and decreased *Per1* and *Ror-α* mRNA expressions were detected in the ND-LL group vs. the ND-LD group. Compared with ND-LD rats, decreased expression of *Clock* mRNA were shown in HFD-LD rats. The expression of *Bmal1*, *Per1* and *Ror-α* were decreased, while *Rev-erbα*, *Cry1* and *Dbp* were elevated in the HFD-LL vs. the HFD-LD group (**Figure 6**).

The Na(+)/H(+) exchanger 3 (NHE3), which responsible for a majority of sodium reabsorption in the proximal tubule, is critical for systemic electrolyte and acid-based homeostasis (11). Expression of *NHE3* mRNA was increasd in the ND-LL *vs.* ND-LD group. Increased *NHE3* expression was observed in the HFD-LL *vs.* HFD-LD group (**Figure 6**).

### ChIP-qPCR assays of BMAL1/CLOCK on *HIF1α* and *Egln1/2* promoters in podocytes (MPC5)

**Figure 7:**
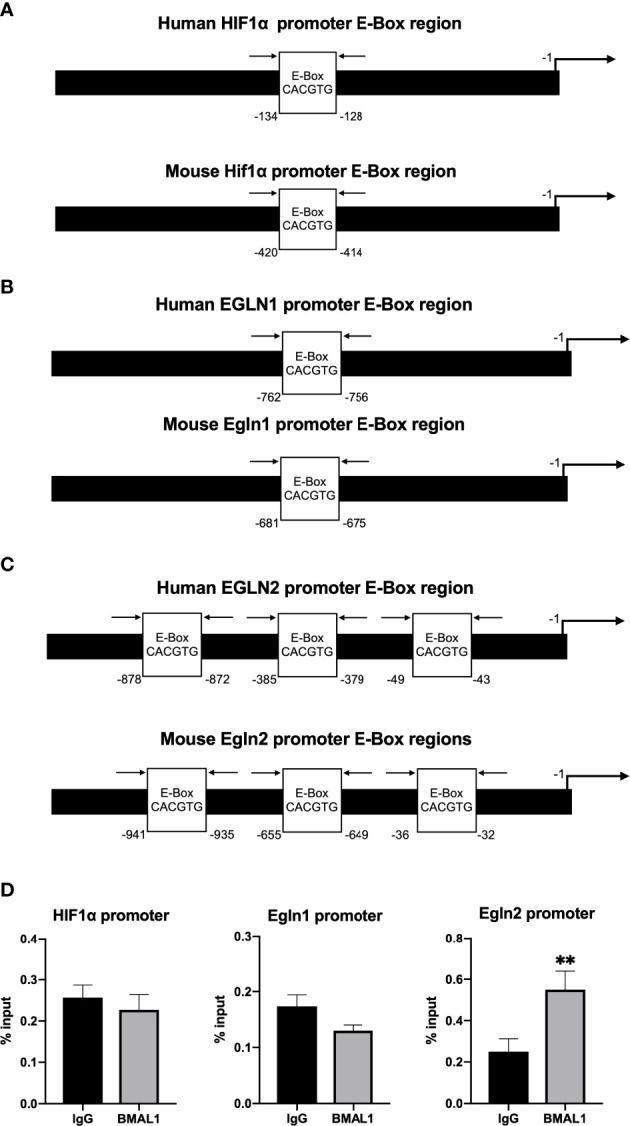
ChIP-qPCR assays of BMAL1/CLOCK on *HIF1α* and *Egln1/2* promoters in podocytes (MPC5). **(A)** Human *HIF1α* and mouse *Hif1α* promoter both contain one E-box. **(B)** Human PHD2 (gene *EGLN1*) and mouse PHD2 (gene *Egln1*) promoter both contain one E-box. **(C)** Human PHD1 (gene *EGLN2*) and mouse PHD1 (gene *Egln2*) promoter both contain three E-boxes. **(D)** Enrichment of BMAL1 was evaluated by quantitative PCR of immunoprecipitated DNA compared with input DNA (% of input). Immunoglobulin G (IgG) was used as a negative control. Values represent mean ± SEM. Differences were determined using two-tailed Student t-test. **p<0.01.

To determine whether or not there is a direct transcriptional regulation of HIF1α and Egln1/2 by circadian clock molecules, we looked for potential enhancer boxes (E-boxes) in their promoters. Human and mouse HIF1*α* (**Figure 7A**), Egln1 (**Figure 7B**) and Egln2 (**Figure 7C**) promoters contain one to three E-boxes. To test if BMAL1/CLOCK binds to any of these boxes, we conducted ChIP-qPCR assays in MPC5 cells. The ChIP-qPCR results revealed that BMAL1 bound directly to the promoter of PHD1 (*Egln2*) (**Figure 7D**).

## Discussion

With growing adoption of artificial light sources, light pollution is increasing by approximately 6% per year worldwide (34). Consequently, light pollution is expected to rise dramatically in the next several decades through more urban development, such as street lightening, security lightening, and vehicles lighting (35). However, consequences of light pollution remain largely unknown. Obesity is a major risk factor for renal disease progression and can cause *de novo* glomerulopathy (4). In the present experiment, we found that chronic constant light exposure potentiates progression of HFD-induced obesity and renal injury in rats. Compared with HFD-LD rats, HFD-LL rats have more severe renal dysfunction, proteinuria, glomerulomegaly, renal inflammation, and glomerulosclerosis. It suggests that light pollution is a novel risk factor for the development of obesity-related renal injury. To the best of our knowledge, this is the first report that links light pollution to kidney diseases.

In normal chow fed rats, we did not observe obvious effects of constant light exposure on proteinuria, glomerulopathy and renal fibrosis. This coincides with the notion that adverse effects of light pollution become more apparent if the animal is challenged with second physiological insult. For example, we recently showed that HFD-fed rats exposed to constant light exposure had exacerbated inflammation and steatohepatitis (17). Similarly, when challenged with constant light exposure, ApoE^-/-^mice exhibit exacerbated dyslipidemia and atherosclerosis (36). However, our study in ND-fed rats found that the ND-LL group had higher TC levels, blood glucose, serum IL-6 and renal CD68 positive cells infiltrations, which may indicate detrimental effects of constant light exposure on metabolisms and chronic inflammation status. Also, glomerular hypertrophy and slightly higher glomerular injury scores may suggest early kidney injury in ND-LL rats.

The link between visceral adiposity and renal diseases is well-established. In a cohort of over 20000 adult participants in the Reasons for Geographic and Racial Differences in Stroke (REGARDS) Study, higher waist circumference was significantly associated with increased risk of developing ESRD, with those in the highest category of waist circumference demonstrating a four-fold higher hazard rate after adjusting for body mass index (BMI). However, after adjusting for waist circumference, no association between BMI and ESRD incidence was apparent. It suggested that central adiposity lies in the causal pathway between obesity and CKD (37). In the present study, we found that HFD-LL rats have more severe visceral adiposity. We found a consistent, significant positive correlation of visceral adipose mass with proteinuria and glomerular injury score in HFD-fed rats. Visceral obesity negatively impacts kidney function directly and indirectly, with the latter resulting from associated complications, such as hypertension, diabetes, hyperlipidemia. More importantly, adipose tissue could release a series of adipokines, such as TNF-α, IL-6, IL-1β, and promote chronic low-grade inflammation in obese patients (38). Our study found that constant light exposure significantly upregulated proinflammatory cytokines including TNF-α, IL-6, and IL-1β in HFD-fed rats. Renal fibrosis is linked tightly to inflammation. In this regard, TNF-α drives the activation of profibrotic cytokine TGF-β and accumulation of extracellular matrix in diabetic nephropathy (39). Paralleled with renal fibrosis progression, we showed a concomitant increasing expression of TGF-β and CTGF in HFD-LL rats as well.

Studies suggest that activation of HIF1α signaling pathways play a pivotal role in renal fibrosis in various kidney diseases (40). For example, Kimura and colleagues found that injection of a pharmacologic HIF1α inhibitor decreased renal fibrosis in unilateral ureteral obstruction model (41). In rat angiotensin II-induced renal injury and chronic ischemic renal injury, the increase of fibrotic proteins (α-smooth muscle actin and collagen) was blocked by HIF1α *sh*RNA (42). Furthermore, HIF1α has been demonstrated to drive the expression of the pro-fibrotic cytokines TGF-β and CTGF during hypoxia (43). Likewise, we observed accumulation of HIF1α protein associated with enhanced TGF-β and CTGF expression in HFD-LL rats which may explain the aggravation of renal fibrosis as a result.

HIF1α is regulated *via* both transcriptional and post-translational mechanisms (44). In ND-fed rats, increased HIF1α expression in ND-LL rats was found only at protein level, while in HFD-fed rats, HIF1α expression was increased at both mRNA and protein levels in HFD-LL rats. It suggests that constant light exposure (light pollution) affects HIF1α thought different mechanisms under ND and HFD, *i.e.* constant light exposure increased HIF1α at post-translational level in ND-fed rats, while in HFD-fed rats, at both transcriptional and post-translational levels.

At transcriptional levels, HIF1α gene has been reported to be directly regulated by molecular clocks (45). HIF1α gene has an E-box at its promoter region and the direct binding of BMAL1/CLOCK to the HIF1α promoter has been reported by ChIP assays in U2OS cells (45). However, in Huh7 cells, CLOCK overexpression had no effect on HIF1α levels (46), indicating that the transcriptional effects of molecular clocks on HIF1α may be cell or tissue-type dependent. In the present study, in a podocyte cell line (MPC5), the ChIP assays did not demonstrate a direct binding of BMAL1 to HIF1α, but revealed that BMAL1 bound to the promoter sites of PHD1, one of HIF1α hydroxylases. It indicates that molecular clocks regulate HIF1α post-translationally at the level of protein stability in podocytes. Translational mechanisms of constant light exposure on renal HIF1α in HFD rats need further study. Constant light exposure sometimes is inevitable, e.g. shift workers, medical staff, et al. Thus, HIF1α inhibition, through activation of PHDs, can be used as an intervention therapy for ORG progression caused by circadian disruption.

Although there are important discoveries revealed by our studies, there are some limitations to note. Firstly, we only used male SD rats in our experiment. There are sex differences in prevalence, risk factors, and mechanisms of CKD (47), highlighting the importance of sex differences in kidney diseases. Yet, the effects of light pollution on female subjects is unknown. Secondly, we only detected renal expressions of clock genes at the time of sacrifice. Our findings suggested circadian disruptions, but rhythmic changes of renal clock genes, as well as changes in daily activity rhythms, may be more reliable assessments for circadian disruptions (48). Thirdly, our experiments indicated that HIF1α signal pathway was involved in the detrimental effects of constant light exposure. However, constant light exposure could promote progression of renal injuries in multiple mechanisms and pathways. Indeed, it has been reported that chronic light exposure influences blood pressure and cause changes of renin-aldosterone system (RAS) (49), which may also affect kidney functional parameters profoundly. Also, the involments of HIF1α pathway do not necessarily imply a direct cause and effect relationship. Further studies are needed to clarify these issues. Fourthly, the light intensity used in our study was 200 lux and the illumination wavelength were 400∼560 nm. Different experimental results may occur when the light intensity changes. Wavelength composition varies throughout the day and studies have shown that spectral variations have very distinct impacts on different circadian, behavioral and physiological responses (50). This suggests that illumination with different wavelengths may have varying effects.

## Conclusion

In conclusion, lifestyle factors associated with circadian disruption and obesity are becoming commonplace in today’s societies. For the first time, the present study demonstrates that circadian disruption by constant light exposure promotes progression of HFD-induced renal injury in SD rats, and provides a potential mechanism in which HIF1α signaling pathway is involved. This data may have implications for devising novel strategies for prevention and treatment of obesity-related kidney disease in the future.

## Data availability statement

The original contributions presented in the study are included in the article/**Supplementary material**. Further inquiries can be directed to the corresponding author.

## Ethics statement

All animal experiments were approved by the Medical Ethics Committee of Central South University and performed in accordance with the guidelines established by the committee (No.2018sydw184).

## Author contributions

LX, SW, YS, RR and DZ contributed to the overall concept and experimental design and reviewed the manuscript. LX, SW, YS, FY, LW conducted the experiments. LX, RR, DZ contributed to data interpretation and edited the manuscript. All authors have read and agreed to the published version of the manuscript.

## Funding

This research was supported by the National Natural Science Foundation of China (NO. 82070884, 81670788), Changsha Science and Technology Bureau Project (NO. kq2004082), and Natural Science Foundation of Hunan province (2021JJ31123).

## Conflict of interest

The authors declare that the research was conducted in the absence of any commercial or financial relationships that could be construed as a potential conflict of interest.

## Publisher’s note

All claims expressed in this article are solely those of the authors and do not necessarily represent those of their affiliated organizations, or those of the publisher, the editors and the reviewers. Any product that may be evaluated in this article, or claim that may be made by its manufacturer, is not guaranteed or endorsed by the publisher.
