# Supplementary material for: Chronic constant light exposure aggravates high fat diet-induced renal injury in rats
Source: Front Endocrinol (Lausanne). 2022 Jul 29;13:900392. doi: 10.3389/fendo.2022.900392 (PMC9372432; doi:10.3389/fendo.2022.900392)
Supplement: Supplementary file 1 [file Table_1.docx]

**Supplementary table 1**

**Primers sequences for Realtime PCR**

| Gene | Forward primer (5’-3’) | Reverse primer (3’-5’) |
| --- | --- | --- |
| TNF-α | ACTCCCAGAAAAGCAAGCAA | CAGTTCCACATCTCGGATCA |
| IL-6 | TCCTACCCCAACTTCCAATGCTC | TTGGATGGTCTTGGTCCTTAGCC |
| IL-1β | TGACCCATGTGAGCTGAAAG | AGGGATTTTGTCGTTGCTTG |
| HIF1α | AGAACTCTCAGCCACAGTGC | CAGAAGGACTTGCTGGCTGA |
| NOX4 | ACTGGTGAAGATTTGCCTGGAAG | CACAGTATAGGCACAAAGGTCCAGA |
| Egln1 | ATTTTGCCAGACCTGTCACC | GCAACACGTCGTCACTCACT |
| Egln2 | CGTGAGGCATGTTGACAATC | CGTCGGTCAGACCAGAAAAT |
| Egln3 | TGACTGCAACTGGCTGGTAG | GCTGCTTGTGGGATTCTAGC |
| Clock | CGGACACGCATGATAGAAGC | AGCATTCACCCCACTCTGAA |
| Bmal1 | TGAACCAGACAATGAGGGCT | TATGCCAAAATAGCCGTCGC |
| Rev-erbα | TGCGGGAGGTGGTAGAATTT | TCGCTGAAGTCAAACATGGC |
| Cry1 | TACAGCAGCCACAAACAACC | AACTGCATGTCGGGCTAGAT |
| Per1 | GTCACCTGCTACCTTCCCTT | TGGGGAACTGCATCTTGAGT |
| Ror-α | GACGGAACTGCATGATGACC | AGTCTCTCCGTTGGTGAAGG |
| Dbp | GCGAGAAGTGCAAAATTGGC | CAAATCCTACGAGCACTGCG |
| NHE3 | GGCCAAGATCGGGTTCCAC | CCCAGGTTGCCGAAGAAGA |
| GAPDH | GACAACTTTGGCATCGTGGA | ATGCAGGGATGATGTTCTGG |

**Primers used for for ChIP assay**

| Gene | Forward primer (5’-3’) | Reverse primer (3’-5’) |
| --- | --- | --- |
| HIF1α | GAACCCATTCCTCACCCATC | GAGCGGCCTAAAAGTTCTTCTG |
| Egln1 | ACCCGTCATTCTCCTTGC | GCAACACGTCGTCACTCACT |
| Egln2 | CCCTGGAGTTGGTGGAAAG | TGTAGCCCAAGCGGATTTT |
